# Physical, Mechanical and Perforation Resistance of Natural-Synthetic Fiber Interply Laminate Hybrid Composites

**DOI:** 10.3390/polym14071322

**Published:** 2022-03-24

**Authors:** Siti Nadia Mohd Bakhori, Mohamad Zaki Hassan, Noremylia Mohd Bakhori, Khairur Rijal Jamaludin, Faizir Ramlie, Mohd Yusof Md Daud, Sa’ardin Abdul Aziz

**Affiliations:** Razak Faculty of Technology and Informatics, Universiti Teknologi Malaysia, Jalan Sultan Yahya Petra, Kuala Lumpur 54100, Malaysia; snadiabakhori@gmail.com (S.N.M.B.); noremyliamb@gmail.com (N.M.B.); khairur.kl@utm.my (K.R.J.); faizir.kl@utm.my (F.R.); yusof.kl@utm.my (M.Y.M.D.); saa.kl@utm.my (S.A.A.)

**Keywords:** natural fiber, synthetic fiber, hybrid composites

## Abstract

Natural and synthetic fibres have emerged in high demand due to their excellent properties. Natural fibres have good mechanical properties and are less expensive, making them a viable substitute for synthetic fibers. Owing to certain drawbacks such as their inconsistent quality and hydrophilic nature, researchers focused on incorporating these two fibres as an alternative to improve the limitations of the single fibre. This review focused on the interply hybridisation of natural and synthetic fibres into composites. Natural fibres and their classifications are discussed. The physical and mechanical properties of these hybrid composites have also been included. A full discussion of the mechanical properties of natural/synthetic fibre hybrid composites such as tensile, flexural, impact, and perforation resistance, as well as their failure modes, is highlighted. Furthermore, the applications and future directions of hybrid composites have been described in details.

## 1. Introduction

Several industries have driven global warming and climate change crises despite the stakeholders knowing the underlying risks [1]. In general, approximately 30% of the greenhouse gases contributed from the energy sector followed by automotive industries that generate the second-most environmental pollution in the form of non-recyclable materials [2]. In order to reduce industrial waste, many manufacturers have been obliged to adopt sustainable manufacturing solutions such as advanced treatment of improper effluent and utilising biodegradable materials. Due to this concern, the trend in automotive technology is moving towards replacing synthetic polymers with biodegradable materials for the components of their vehicles [3]. Green materials like natural fibre are gaining popularity for commercial applications due to their advantages such as low-density, lower cost, and comparable specific strength to weight ratio as compared to conventional materials [4,5,6,7,8]. 

Recently, according to Kurien et al., global natural fibre production has steadily increased, making these fibres a viable source for producing composite materials. During the projected period, the composite market is estimated to increase at a compound annual growth rate (CAGR) of 15.0%, from USD 436 million in 2018 to USD 876 million in 2023. As a result, the development of composites with environmentally fibres is extensively being developed [9].

The fundamental problem with using natural fibres in structural composites is the inconsistency of their mechanical characteristics and their hydrophilic nature [10,11]. They could absorb a lot of moisture, poor matrix-fiber interface adhesion and very combustible, which limits their use in polymer reinforcement [12]. To overcome this issue, in recent years, composite hybridized techniques via combining two or more reinforcements into a matrix have been focused on. The major goal of this work is to improve the limitations of a single-fiber reinforced matrix with qualities that are similar to or better than the original composite [13]. For example, substituting carbon fibres in the centre of a laminate with less expensive glass fibres can drastically reduce the cost while retaining almost all of the flexural capabilities. When a hybrid composite is loaded in tension in the fibre direction, the more brittle fibres fail first, followed by the stronger fibres [14]. 

Also, combining natural fiber with synthetic fibre can increase their mechanical performance, moisture absorption resistance, and most importantly, balance the environmental effect of the waste composite materials [15]. This hybrid reinforcement could widely range from blending the short fibre in a matrix that are randomly oriented to woven fibres laminates arranged in particular directions. Also, the interlacing of fibre bundles, on the other hand, could improve the strength of composites, increases its damage tolerance, and offers a net form structural component.

In this review the classification of natural, synthetic, and composite fibres was initially discussed. In the subsequent subtopics, the positive effects of combining the natural-synthetic fibre were also evaluated. Then, physical and mechanical properties of interply composite laminates were clarified in detail. Finally, a perforation resistance, failure mode, applications, and future directions of interply hybrid composites were briefly explained.

## 2. Natural and Synthetic Fibers

Natural fibres are classified into three major groups: animal, mineral, and vegetable fibre as shown in Table 1. 

In animal fibres, protein is a common substance in the animal fibre. It can be grouped into sub-classes of wool, silk, human hair, and feathers. Wool is distinguished from animal hair and fur by various characteristics, like being crimped, stretchy, and growing in staples. For example, sheep wool, goat hair, alpaca hair, and horse hair are predominantly fibres that are used in the textile industry [17]. The location of these dairy animals, which are grown in a broad diversity of heights and temperatures, influences the mechanical properties of these fibres [18].

In addition, asbestos is classified as a modified mineral fibres. Asbestos is a silicate compounds having a silicon and oxygen chemical structure that occur naturally in the form of fibre bundles in the environment. These fibres are resistant to heat and fire, as well as being poor conductors of electricity [19].

Plant fibres are widely recognised by the industry and the most extensively studied by researchers. This is due to the short growth period, renewability, and broader availability of the product. Cellulose, hemicellulose, and lignin compensate the vegetable fibres. They can be collected from bast, leaf, seed, fruit, wood, stalk, and grass/reed. 

Bast fibre was collected from the inner bank or it could be obtained from the surrounding stem of the plant. When compared to other natural fibres, these fibres have a higher tensile strength [20]. Leaf fibre is a stiff, coarse fibre derived from monocotyledonous plants’ leaves. Leaf fibres are mostly used for cordage such as rope and twine and woven fabrics as well. Seed fibres are soft, cool, known as breathable fibers, and absorbent extracted from the seeds of different plants [21]. Wood-based fibres are derived from trees with a lignin-rich, woody trunk that is formed by secondary growth. The lignin content of wood fibres is often higher than that of non-wood fibres [22]. For stalk fibres, they cultivated from plant straws such as wheat, oat, maize, barley, or rape and grass/reed fibers can be discovered in the ground and vascular tissues [23].

Since other fibres have limitations, most studies concentrate primarily on vegetable fibres. Animal fibres are rarely utilised, while mineral fibres such as asbestos are prohibited owing to the risk of human health exposure [24]. Natural fibers from bast fibers such as flax, kenaf, and hemp are the most often utilised natural fibres in industrial applications. 

Natural vegetable fibres are primarily composed of cellulose, hemicellulose, and lignin, with waxes and other compounds occurring in lesser amounts. Natural fibres chemical composition varies by species and determines their fiber properties. Table 2 shows the chemical composition of the principal fibres used as reinforcement in composites.

Cellulose: Cellulose makes up the majority of natural fibres. The physical qualities of natural fibres are regulated by hydrogen bonding, which determines the crystallinity of cellulose. It is the key component that gives them strength, rigidity, and stability [31].

Hemicelluloses: Hemicelluloses are polysaccharides linked together in short, branching chains. They are closely linked to cellulose microfibrils and help embed the cellulose in a matrix. Hemicelluloses are naturally hydrophilic and their molecules have a lower molecular weight than cellulose [22].

Lignin: It’s a complex aromatic hydrocarbon polymer that gives plants their stiffness. Different plant species have various lignin compositions, as do different tissues within the same plant. Without lignin, plants would not be able to reach great heights. Lignin is a three-dimensional polymer that is less polar than cellulose, has an amorphous structure, and has a larger molecular weight. It functions as a chemical adhesive within and between fibres [32].

The structure of fibres, as well as their physical and chemical content, which are the most important elements in defining the overall qualities of fibres, are strongly affected by the age of the plant, species, climate, harvesting time, and fibre processing techniques [33].

The tensile strength and elastic modulus of several natural and synthetic fibres are listed in Table 3. These figures suggest that natural fibre qualities are equivalent to, or even superior to those of glass fibres in some circumstances. Natural fibre mechanical characteristics will be influenced by a number of factors. Processing techniques for fibre extraction, aspect ratio, cultivation conditions, matrix selection, interfacial strength, fibre distribution, stacking sequence, composite production process, and permeability are the primary elements impacting mechanical performance [34].

Utilising natural materials through modern tools and technical knowledge has resulted in exciting developments in the composite field [45]. The summary of advantages and disadvantages of natural fibre composites as compared to conventional petrochemical-based composites is presented in Table 4. 

Synthetic fibres were the first fibres used as reinforcement in composite materials [47]. They are made from petroleum by-products and are divided into organic (aramid, polyethylene, and polyester) and inorganic types (glass, carbon, boron, and basalt) [48]. The most common synthetic fibers used in the composites industry are glass fibres, carbon fibers, and Kevlar (aramid). The physical and mechanical properties of glass, carbon, and aramid fibers are tabulated in Table 5.

In advanced composites, aramid fibre with high tensile modulus and strength was the first organic fibre used as reinforcement. Heat resistance, low flammability, and excellent resistance to organic solvents are all advantages of aramid fibres. The fibres are lightweight, strong, and tough, making them very useful in aeronautics. Such as Kevlar 49, which exhibits high stiffness, and Kevlar 29, with low stiffness [50].

Glass fibre products now account for more than 95% of the fibre reinforcements used in composite industries [51]. Compared to metals, it has superior bulk strength, stiffness, and lightweight properties [49]. The common types of glass fibres for mechanical engineering applications are S-glass and E-glass fibres. S-glass fibres are stiffer and stronger than E-glass fibres and have better resistance to fatigue and creep [52]. However, E-glass fibres are frequently used among all fibrous reinforcements due to their low cost [53]. 

Carbon fibre is composed of a continuous chain of carbon atoms linked together [54]. They are used broadly in a range of applications, including aerospace, chemical industries, general engineering, missiles, nuclear energy, and textiles [55]. It is lightweight and offers excellent impact resistance, as well as being one of the strongest and stiffest commercially available fibre reinforcements for composite structures [56,57].

## 3. Hybrid Composite Laminates

**Figure 1:**
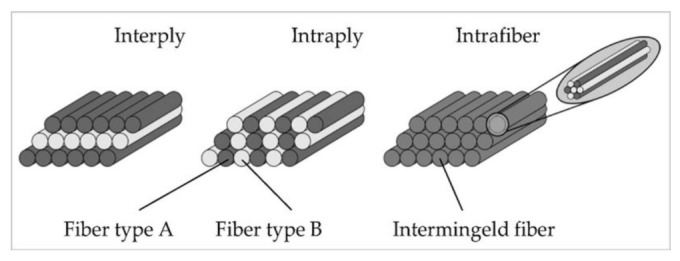
Hybrid configurations for continuous and discontinuous fibre reinforced composites: interply, intraply and intermingled adapted from [62] MDPI, 2022.

In hybrid composites, the mechanical properties of hybrid composites are stacking sequence dependent [58]. Mechanical properties such as flexural and impact properties might vary depending on the stacking order of fibre layers for the same volume fraction of fibre components [59]. Hybridisation, for example, was discovered to provide composites with the highest load resistance and energy absorption [60]. The types of hybrid composites are differentiated based on fibrous reinforcement, which includes: (1) layered (interply), (2) interwoven (intraply), and (3) intermingled, as shown in Figure 1. Each intraply reinforcement, whether in the shape of a fabric or a mat, is made up of multiple different types of fibres [61]. Intrafibre reinforcement is made up of a variety of fibres that are mixed and hybridised within the ply. Interply, on the other hand, is made up of layers of individual reinforcements in the shape of textiles or mats that are piled together.

In interply, each reinforcement in the form of fabric or a mat is made up of one type of fibre. To get the optimum hybrid mechanical characteristics, the fibres were then laid up in alternative sequences and directions [63]. Choosing a high-strength fibre will result in a hybrid with the highest mechanical strength [64]. The sequence of their arrangement is also crucial. The impact energy is absorbed by the initial layers, which are made of a strong shear-resistant material [65]. Tensile-resistant fibres should be used in the middle and rear layers. The volume fractions should be encountered as the volume percentage of the dominant reinforcement increases, the flexural and tensile strengths of the composite increase [64]. The physical and mechanical characteristics of interply hybrid composites are influenced by all of these aspects. Interply hybridization has been successfully exploited by several researchers to improve the mechanical characteristics of composite laminates [60,62,66,67,68].

## 4. Physical Properties of Interply Hybrid Composites

Fibre reinforcements’ physical and mechanical qualities are largely determined by their physical composition. The structure of natural fibres, cellulose concentration, fibril angle, and cross section are all important. They can vary significantly according to the production environment, origin place, and other factors. Synthetic fibres, on the other hand, are determined by the chemical content of the fibre’s shape, size, and strength.

### 4.1. Thermal Analysis

Thermal stability, thermal resistance, durability, and heat resistance are often described by thermal stability. In the current scenario, polymer-based industries show a growing interest in obtaining polymers with increased thermal stability [69]. Thermal studies are also involved in determining the thermodynamic properties of various developed composites, including for crystallisation analysis, glass transition temperature (T_g_), and viscoelastic mechanical behaviour. Further, Gupta investigated the dynamic mechanical and thermal properties of hybrid jute/sisal fibre reinforced epoxy hybrid composites. The thermal properties of the hybrid composites had higher values of glass transition (T_g_), crystallisation temperature (T_c_), and decomposition temperature (T_d_) than epoxy, which showed a positive effect of reinforcement of jute and sisal fibres [70]. The thermal analysis obtained from several studies is shown in Table 6.

### 4.2. Fibre Volume Fractions

Naveen et al. developed high performance structural composites using Kevlar/cocos-nucifera reinforced epoxy hybrid composites. The results revealed that hybrid composites with weight ratios of 75% Kevlar and 25% cocos-nucifera offered a virtuous resistance or stability towards heat in the epoxy composites [72]. An experimental work aimed to study the effect of weight fraction on the mechanical properties of flax and jute fibres reinforced hybrid composites by compression moulding method was carried out by Karthi et al. It was observed that the maximum tensile strength, flexural strength, interlaminar shear strength, and impact strength were obtained only for a composite having 30 wt% flax fibre reinforcement [80]. Ismail et al. fabricated the hybrid composites of kenaf/bamboo fibre mat-reinforced epoxy hybrid composites. Kenaf, bamboo, and kenaf/bamboo hybrid composites were prepared by using the hand lay-up method at 40 wt% total fibre loading. The findings showed that 50:50 ratio gave the highest flexural and impact strength [81].

### 4.3. Water/Moisture Absorption and Swelling

The moisture absorption of composites containing natural fibres has several adverse effects on their properties; hence, it can cause an increase in moisture (leading the fibres to swell), a decrease in their mechanical properties, provide necessary conditions for biodegradation, and change their dimensions [82]. Natural fibre reinforced composites have low durability and inherently absorb a lot of moisture. This can compromise their characteristics and, consequently, impact their long-term function. Due to that, water absorption affects the compatibility between fibre and matrix, leading to poor stress transfer efficiencies from matrix to reinforcement in hybrid composites [83]. The study done by Ramesh et al. confirmed that the major drawback of any natural fibre and its composites is their moisture absorption nature [84]. According to Karimzadeh et al., the weight percentage of the moisture uptake was calculated using Equation (1) [85]:

(1) $$\mathrm{Weight}\;\mathrm{Percentage}\;(\%)=\frac{M_{t}-M_{0}\;}{M_{0}}\times \;100$$

where $M_{t}$ is the specimen weight at time t of exposure to moisture and $M_{0}$ is the specimen weight before immersion. 

Ravikumar et al. analysed the effect of water absorption behaviours of jute/carbon fibre reinforced polyester hybrid composites. The result showed that all the composites developed followed the Fickian behaviour of water absorption [86]. Elsad et al. studied the effect of water absorption on the tensile characteristics of flax/sisal/carbon/glass fabrics reinforced by unsaturated polyester-based hybrid composites. From the results, they concluded that the tensile strength drop due to water penetration was minor for hybrid composites with synthetic fibres at the outer layer but significant for hybrid composites with natural fibres at the outer layer [87].

It is known that the swelling of composite materials is mainly caused by water uptake. The correlation between water uptake and the thickness swelling of composites was also reported. It is important to study the moisture absorption and swelling behaviour of natural fibre composites to estimate the consequent effects on the performance of composite parts. Nasimudeen et al. carried out an experiment to investigate the effect of water absorption and thickness swelling behaviour of hybridised natural fibres such as banana, jute, and kenaf in different stacking sequences in vinyl ester. They improved the strength, stiffness, and lower moisture absorption properties of the composite laminates [88]. Moreover, Thiagamani et al. reported the water absorption characteristics of sisal/hemp hybrid composites, which exhibited a linear rise in the first days of soaking in water, as well as a greater rate of thickness swelling in the hybrid composites. They found that porosity, fibre-matrix adhesion, vacancy percentage, and lumen size were the key factors influencing water absorption in natural fibre reinforced composites [89]. Ismail et al. evaluated the physical properties of kenaf/bamboo fibre mat-reinforced epoxy hybrid composites. The density, water absorption, and thickness swelling of the composites were reported to have increased as the kenaf weight ratio increased [81]. In other work, Mert et al. developed hybrid composites from jute fibre and wood particles that were manufactured by using the vacuum-assisted resin transfer moulding technique. The results of tests of physical properties showed that the wood/polyester specimen had the lowest values of thickness swelling, water absorption, and moisture content as compared to the jute/polyester and jute–wood/polyester composites [90]. 

## 5. Mechanical Properties of Interply Hybrid Composites

Mechanical properties of hybrid composite can be discussed on the tensile strength, charpy-izod and bending properties. The work on natural-synthetic hybrid composites is shown in Table 7.

### 5.1. Tensile Properties of Interply Hybrid Composites

**Figure 2:**
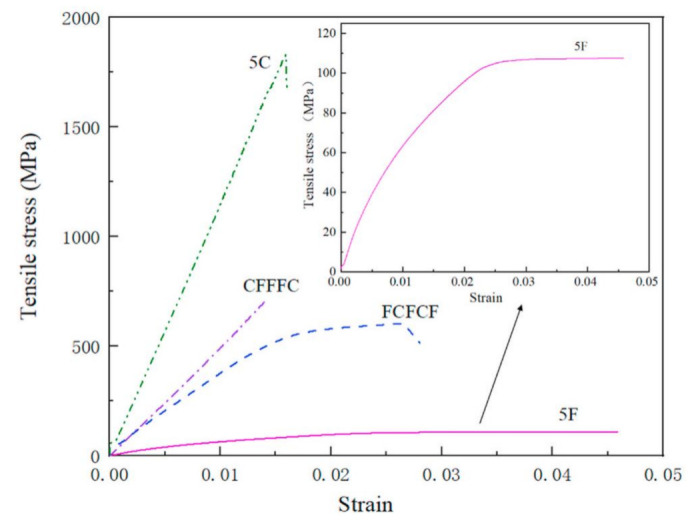
Tensile stress-strain curve of flax/carbon hybrid composite laminates (adapted with permission Elsevier, 2022) [109].

Tensile tests are the most important investigations that predict the applications of materials material [107]. The tensile strength is mainly used to evaluate the strength behaviour of a composite material [108]. A relationship between the load applied to a material and the deformation of the natural-synthetic hybrid composite is expressed through a stress-strain curve as shown in Figure 2, derived from tensile testing. The stress–strain curve is split into two parts: a linear component that reflects the deformation of each cell wall, and a nonlinear part that describes the elastic–plastic deformation of the fibres. The total nonlinearity of the produced composites was reduced by combining natural and synthetic fibres. When high- and low-ductility fibres are hybridised, pseudo-ductility is frequently the result. In comparison to pure natural fibre composites, hybridization of natural-synthetic fibres resulted in an improvement in tensile strength and modulus. Both hybrid systems outperform pure composites in terms of strength and modulus [109].

Selmy et al. explored the possibility of using polyamide (PA) as a reinforcement with glass to form new hybrid composites with improved physical and mechanical properties using the hand layup technique in an inter-ply configuration. These hybrid composites were suitable for medium load applications due to the existence of glass fibre at the composite external layers and PA-fibre in the core improved the tensile and flexural properties but worsened the shear properties. Furthermore, increasing the PA-fiber relative volume fraction upgraded the tensile properties, but deteriorated the flexural and shear properties [59]. Wu et al. investigated the tensile and compressive properties of interlayer and intralayer hybrid composites, finding that experimental tensile and compressive strengths for interlayer and intralayer hybrid composites were higher than theoretical values, indicating that strength conformed well to the positive hybrid effect [68]. 

Hybrid composites have better mechanical characteristics that can overcome the disadvantages of single fibre composites. Rihayat et al. proved that hybrid composites from bamboo, pineapple leaf, and coir are capable of produced high value of tensile strength than the single fiber mixture of coconut coir and palf fibers in the polyester matrix [110].

Khalid et al. conducted experimental and numerical characterization of tensile properties of the jute/carbon fabric reinforced epoxy hybrid composites. They found that an increase in jute percentage reduces the strength of the hybrid composites due to heterogenous jute properties and the waviness of fabrics [101]. Venkatasudhahar et al. have investigated the influence of stacking sequence and fibre treatment on the mechanical properties of carbon/jute/banana reinforced epoxy. Results found similar results to previous research where a hybrid composite with stronger fibre as the outermost layer gives outstanding performance [102]. In another study, Kevlar and date palm reinforced with epoxy were used to fabricate a hybrid composite for tensile and material analysis for automotive applications. They concluded that the stress, strain, and deformation are very close compared to the existing material [106]. Mittal and Chaudhary investigated the development of palf/glass and coir/glass fibre reinforced composites. The results show that combining chemically treated cellulosic and glass fibres in an optimum volume ratio has better properties than the single glass fibre reinforced material [95]. Apart from that, other researchers studied the hybridization of glass/jute hybrid composites through mechanical performance and found that the hybridization of glass/jute hybrid composites increased the mechanical properties with limitations [96]. This investigation is similar to a study done by Ismail et al. where the tensile properties of glass/jute fiber-reinforced composite increase with optimum loading but decrease with further loading of jute fiber. This is caused by poor interfacial bonding between the matrix and reinforcement. Due to an excess amount of jute fibre and fewer matrix causes, the mixing process did not happen equally [98]. Mohammed et al. prepared kenaf/glass fibre hybrid composites to study the weathering effects on the mechanical, morphological, and thermal properties of the pure kenaf hybrid composites. Results obtained showed that the natural fibres and their resultant composites could not withstand environmental conditions due to poor wettability with some polymeric matrices [94]. In the study done by Sathiyamoorthy and Senthilkumar, the mechanical, thermal, and water absorption behaviours of jute/carbon reinforced hybrid composites are affected by the stacking sequence of natural and synthetic fibres. When tested in tensile, hybrid composite laminates with natural fibre as the outer layer show good performance when tested. However, a hybrid composite with synthetic fibre as the outer layer of the laminates exhibits greater impact strength and better moisture resistance [100].

### 5.2. Charpy and Izod Test

**Figure 3:**
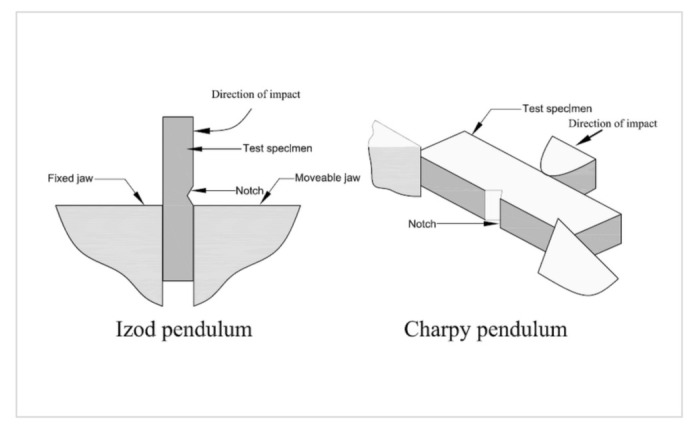
The schematic diagram of izod and charpy impact test (adapted with permission Elsevier, 2022) [113].

The charpy and izod impact tests, were classified as low velocity impact and were used to measure a material’s impact strength and toughness [111]. The approach calculates the amount of energy absorbed during fracture by a notched or unnotched sample of a material. Then there’s absorbed energy, which is a measure of a material’s toughness and may be used to research the ductile-brittle temperature transition [112]. Figure 3 depicts both the effects of Charpy and the izod tests. The difference between the two techniques lies in the manner of specimen support. For the Izod Impact Test, the specimen is placed vertically, whereas in the Charpy Impact Test, the sample is placed horizontally on the specimen holder.

A series of charpy impact tests were conducted by striking either side of the Kevlar/S-glass/epoxy hybrid composite laminates samples, according to Erkli and Bulut, to determine the amount of impact strength and absorbed energy. The degree of hybridisation effects revealed that the use of Kevlar layers instead of glass layers significantly increased the total composite laminate’s impact strength [115]. Wang et al. found that hybrid fibre-reinforced composites made with polyimide fibre and carbon fibre as reinforcement and epoxy resin as the matrix increased Charpy impact strength by approximately 50% when compared to carbon fibre-reinforced composites [116]. Rassiah et al. described a woven bamboo/E-glass epoxy hybrid composite hand lay-up laminate technique. They investigate how alternative bamboo and E-glass weaving sequences influence the mechanical characteristics of hybrid composites [117].

Impact test conducted by Izod test is affected by the rate of loading, temperature and presence of stress raisers, heat treatment processes, and alloy content in the material [118]. A drop-weight impact response of hybrid composites was investigated by making laminates of epoxy-based hybrid composites reinforced with E-glass/Kevlar. E-glass/Kevlar 49 at layup 0°/90°and 30°/60°exhibited improved impact strength more than 45°/45° for Izod test [119]. Da S. Vieira et al. presented a work to evaluate and compare the mechanical properties of composites formed by hybrid fabric with 70% malva—30% jute in epoxy polymer matrix carried out by combination of Charpy and Izod impact tests. Therefore, 30% of the samples had the best results, functioning as an efficient reinforcement [120]. The differences between both tests are shown in Table 8.

Rashid et al. studied the capabilities of coir-Aramid/Epoxy hybrid composites by comparing the impact responses effect of different stacking configurations. The results found that woven coir/aramid-epoxy leads to improve impact properties by modifying the structure of composites and using the layering system [97]. Banu et al. compared the effects of the stacking sequence of Kevlar and natural fibers/epoxy polymer composites by using aloe vera, bamboo, and palm fibers. They concluded that the aloe vera/palm-Kevlar hybrid composite gives the highest impact strength because aloe vera and palm fibres have better wettability with the matrix and synthetic fibers [105].

### 5.3. Flexural Strength

Bending tests are ideal for components that are subjected to bending loads during operation, as both the flexular strength and the flexural modulus can be determined in a simple test with simple sample preparation. This test also shows durability due to loading application of a material is strongly influenced by the physical and mechanical properties of the material [110]. Three-point flexure bending tests and four-point flexure bending tests are the most frequent flexure tests. Wagih et al. investigated the residual flexural strength of a pre-impacted carbon-aramid hybrid composite utilising three-point bending. The carbon fibre plies in the bottom section of the laminate (non-impacted face) were not shattered following the impact test or the three-point bending test, unlike all-carbon/epoxy laminates [121]. Sharma et al. presented a work involving fabrication of hybrid fibre-reinforced polymer (FRP) composites and investigated the change in their flexural strength concerning the stacking sequence and fibre, which was tested under three point-loading. The test showed that the accumulative stacking of carbon and glass laminates gaves better flexural strength as compared to another fabricated specimen [122]. The combined effect of compression on the top ply, tension on the bottom ply, and shear on the middle plies affects the flexural characteristics of composite laminates in general. Fabric plies’ stacking sequences in hybrid laminates had a significant influence on composites’ flexural properties [123]. In this case, Nagaraja et al. studied the effect of stacking sequence on the tensile and flexural properties of carbon-glass/epoxy hybrid composite laminates. Out of the two laminates tested, the one with carbon fabric positioned at exterior regions showed highest flexural strength [124].

The hybrid design strongly affects a variety of properties such as flexural strength, modulus fatigue behaviour, and impact performance of the hybrid composite upon the performance of reinforcement and matrix. In addition, the mechanical properties of hybrid composites strongly depend on the reinforcing fibre position [125]. Bending test results of hybrid composites of drumstick fibres, glass fiber, and polyester resin revealed transverse fibre orientation that sudden fracture took place due to fibre failure and matrix collapse in composites [126]. In other cases, the flexural strength of the composite increased with an increase in the fibre content of polymer hybrid composites made by reinforcing jute, pineapple leaf fibre, and glass fibre in a 1:1:1 ratio into an epoxy resin, as studied by Reddy et al. [127]. Furthermore, the flexural strength of the jute/carbon-epoxy hybrid composite was comparable to that of the carbon-epoxy composite and much higher than that of the jute-epoxy composite [128]. Moreover, Jesthi and Nayak stated that the flexural strength was improved by 55% in comparison to a plain glass fibre-reinforced polymer composite in dry conditions [58]. 

Mat Jusoh et al. examined the effects of stacking sequence on the tensile and flexural properties of glass fibre epoxy composites hybridised with basalt, flax, or jute fibers. The result proved that the arrangement of fibres in hybrid composite laminates enhanced the flexural strength of the fibers [91]. Jamal et al. studied the effect of the structural integrity effect of interleaf glass mat and recycled GFRP waste on woven kenaf reinforcement. Polyester composites with fibre volume fraction were kept constant. Results showed that the flexural strength improved by up to 47.5% and indicated that the recycled GFRP could replace glass fibre as reinforcement [92]. Abd El-baky and Attia investigated the tensile and bending performances of hybrid jute-glass-carbon epoxy composites with different layer configurations. They showed that the layer configurations have no effect on tensile properties; however, the composites with carbon as the outermost layers exhibit maximum flexural properties [129]. This study was supported by Santhanam et al. that the stacking sequence had a negligible effect on the tensile properties [130]. In contrast, the flexural and impact strength were largely affected by the woven glass fiber and banana fiber stacking arrangement. The effect of stacking sequence and ply orientation on the mechanical properties of hybrid composites has been widely investigated.

## 6. Perforation Resistance of Hybrid Composite

Low-velocity impacts are referred to as impacts with speeds of between 1 m/s and 10 m/s. It is determined by target stiffness, material qualities, and projectile mass and stiffness, and has recently received a lot of attention. The low-velocity impact was used to investigate how a material reacts when subjected to a drop-weight impact at various energy levels. The force displacement curve, which illustrates the impact behaviour of a material when subjected to a low-velocity impact, is a common representation of a material’s impact properties. The open curve and closed loop patterns are the two varieties. The closed-loop type curve depicts penetration and rebound impactors, whereas the open-loop force-displacement curve depicts the complete perforation of the sample. According to Sunith et al., impact velocities can be divided into four categories for testing purposes: low velocity impact (drop testing and pendulum testing), intermediate velocity impact, high velocity impact (ballistic testing), and hyper velocity impact [131].

According to the principle of conservation of energy, the energy absorbed by the sample is equal to the energy produced to cause the damage. The notion of drop weight is inspired by the first law of vibrations; the static mode is used in low velocity collisions. The absorbed incident energy in composites might result in extensive fracture regions. As a result, the composite’s strength and stiffness are lowered. The time it takes for the impact wave to reach its limit and return to its original position is longer than the contact force duration. When the force curve reaches zero, maximum deflection occurs. This phenomenon is also known as an energy balance model, in which the system’s overall energy is conserved while higher vibration modes, friction, and other energy losses are ignored. Differences in low velocity impact response and impact damage are directly related to differences in sample dimensions and manufacturing processes. Table 9 shows the study on the low velocity impact performance of hybrid composites.

Jusoh et al. evaluated the indentation and low velocity impact behaviours of homogeneous and hybrid composites of woven E-glass with basalt, jute, and flax. For hybrid composites, the impact test revealed a higher peak force, whereas the indentation test showed maximum deflection [91]. The low-velocity impact performance of glass fibre, kenaf fibre, and hybrid glass/kenaf fibre composite lamination was investigated by Majid et al., and it was discovered that hybridisation can improve load-carrying capacity under impact loading [5]. The quasi-static penetration behaviour of plain woven kenaf/aramid-reinforced polyvinyl butyral hybrid laminates was shown to be positive in terms of maximum load carried, energy absorbed in impact, and damage processes [138]. Md. Shah et al. studied the hybridisation of bamboo and glass-reinforced epoxy composites. The use of woven glass fibre in composite laminates could slow the impactor’s penetration, reducing the likelihood of the composites failing completely [153]. Chen et al. investigated the effects of hybridisation of a carbon/glass/basalt-reinforced composite on the low velocity impact resistance. In terms of damage area, full hybridization of three fibre types allows for more global deformation of composite laminates. When compared to the identical stacking arrangement using carbon and glass or basalt fibres, the placement of carbon layers as the core obtained the maximum energy absorption performance [141]. Impact studies revealed that natural or hybrid composites absorbed more impact energy than glass/epoxy composites due to substantial impact damages, according to Selver et al. [150]. 

Ismail et al. investigated the effects of combining kenaf with glass fibre to create hybrid composites with varied weight ratios. Since the combination of 75% glass fibre and 25% kenaf fibre had the best tensile qualities, it was chosen to be tested for its low velocity impact properties in the study. The hybrid composites were found to be able to endure impact energy up to 40 J with the highest impact load, and the absorbed energy increased as the incident impact energy rose [142]. Al-hajaj et al. characterised the impact properties of a new hybrid composite made with woven carbon fibres plus unidirectional or cross-plied flax fibres in an epoxy matrix. The findings showed that cross-plied fibres had better impact performance [140]. 

In particular, there are factors that have a significant influence on low velocity impact performance. Bandaru et al. conducted an experiment and a numerical investigation into the low velocity impact response of Kevlar and basalt reinforced polypropylene. They stated that the type of hybridisation and stacking sequence of the layers greatly influences the impact performance of the composites. The alternative stacking sequence of hybrid composite enhanced the impact performance by up to 20% in terms of peak force and anergy absorption [63]. Nor et al. investigated the tensile and impact properties of hybrid composites based on kenaf, jute, and fibreglass woven fabrics subjected to low-velocity impact and concluded that an alternate sequence of hybrid composites exhibited more impact resistance [139].

Subramaniam et al. studied the effects of stacking configuration on the response of tensile and quasi-static penetration to woven kenaf/glass hybrid composite metal laminate. Hybrid laminates had a 15% improvement in energy absorption and were able to distribute contact stress, while the middle layer operated as a crack propagation barrier [143]. Prasath et al. illustrated the combinations of flax/basalt hybrid composites. The overall performance of the alternate arrangement of basalt and flax fibre composite for low velocity impact and compression following impact experiments is better [145]. Feng et al. compared the indentation behaviour of hybrid composites to that of non-hybrid composites by looking at the effects of different fibre stacking configurations on the indentation behaviour of pineapple leaf/glass fibre reinforced polypropylene hybrid composites. Indentation resistance and energy absorption were comparable between the hybrid glass fibre-based composites and the non-hybrid glass fibre-based composites when the central glass fibre was replaced with pineapple leaf fibre [146]. Similar to the study done by Zulkafli et al., glass fibre reinforcement in banana fibre reinforced composites increases energy absorption and improves overall impact performance [147]. Mahesh et al. examined the stacking sequence configuration of flexible green composites for cladding application under a low velocity impact regime. Through the standalone FE simulations, an alternate sequence of hybrid composites shows a higher peak force [157]. Because of the ability to better contain damage in a restricted area, the use of different stacking sequences provided a better impact energy absorption capability [158].

To get the most out of these advanced material systems, a thorough understanding of the interactions between the various phases in the hybrids is required. Malingam et al. studied the different fibre configurations of composite laminate materials and found that the penetration resistance of composite laminates is governed by the bending stiffness of each fibre ply. Therefore, the placement of Kevlar fibre in the surface layers improved the penetration resistance of the laminates [137]. Similar to work done by Wang et al., the energy absorption of samples with higher stiffness fibre as the outer layer was greater compared to hybrid composites with an optimum stacking sequence [109]. In their study on carbon/flax epoxy, Ravandi et al. stated that when compared to a non-hybrid flax composite, transferring the carbon plies to the impact side had no effect on impact resistance but did result in a 10% reduction in the impact perforation threshold [144]. Paturel and Dhakal examined low velocity impact behaviour of flax/glass hybrid laminates. With a glass fibre layer on the top and another one on the rear, the load bearing capability increased by 25% [151]. This is because the outermost surface had higher energy absorption, which made it more impact resistant than other composite samples [148]. This was supported by Gemi where the maximum contact force, maximum displacement, contact duration, and absorbed energy values all increased with increasing impact energies [136]. Fragassa et al. performed an analysis of the mechanical and impact properties of flax and basalt fibres and their hybrids. The findings suggest that more complex stacking sequences incorporating intercalation of flax and basalt layers may be adopted in the future [135].

Fiore et al. studied the ageing resistance of jute and jute-basalt interply hybrid laminates exposed to salt-fog with the goal of investigating the possibilities of using a ply-substitution strategy to improve the durability of natural fibre reinforced composites for maritime applications. The results of environmental ageing revealed lower decrements of the impact peak load (3.5%) compared to jute laminate, which has a value of 10.5% [133]. ivkovi et al. evaluated the impact properties of flax, basalt, and hybrid flax/basalt fibre reinforced green composites. It was concluded that there were significant differences in the impact behaviours of dry and conditioned single-type composites [134]. Amir et al. evaluated the low velocity impact and compression after impact properties of gamma-irradiated Kevlar/oil palm empty fruit bunch hybrid composites. When the hybrid composites were treated with gamma radiation, there was no substantial improvement in impact resistance [159]. The link between the degree of impact damage and natural frequency was highlighted by Chok et al. They found that as the impact level rose, the natural frequency reduced [132]. The influence of employing multi-walled carbon nanotube material (MWCNT) as nanofillers in low-velocity impact (LVI) followed by ultrasonic wave propagation imaging (UWPI) to visualise the affected damage and compression after impact (CAI) properties of bamboo/glass fibre hybrid composites was investigated by Farhan et al. [149]. The presence of CNTs absorbed less energy than neat bamboo/glass hybrids, resulting in less physical damage. In the study of impact damage resistance of jute/Kevlar hybrid composite laminates subjected to varying heights by Bhanupratap, it was concluded that the dynamic response of these frameworks was dependent on the flexibility of the fibre material [152]. The temperature effect on the single and repeated impact responses of intraply flax/basalt hybrid polypropylene composites was studied by Ferrante et al. Decreasing temperatures caused embrittlement in neat PP composites, resulting in an increase in maximum force and a decrease in maximum displacement, whereas increasing temperatures caused compatibilised composites to soften, resulting in a decrease in maximum force and an increase in maximum displacement [155]. In the study by Basha et al., the damage mechanisms of CFRP/wood sandwich laminates with various wood core types were explored under low velocity impact and CAI. They stated that wood cells deformed during impact and hence dissipated more energy. From past research, it is clear that a variety of factors influenced the outcome of hybrid composites’ low-velocity impact performance [156].

## 7. Failure Mode of Perforation Resistance on Hybrid Composites 

Failure of laminated composite plates has been a major concern for many years, and various studies have been undertaken on the subject. Fibre failure in tension, matrix failure in tension, fibre failure in compression, matrix failure in compression, and delamination are all examples of laminated composite failure modes [160]. Impact causes the most serious damage, such as holes and fissures, which can impair strength. Impact failure can affect a number of circumstances, such as fibre/matrix adhesion, reinforcement characteristics, thickness, and matrix properties. Almost every hit weakens the material, reducing its stiffness and strength [142]. The impact energy delivered to the composite determines the damage behaviour. The damage behaviour of composite materials, on the other hand, is highly dependent on each of the constituents in the composite. The damage to the composites is caused by the impact reaction, which can be elastic, plastic, or fluid, or any combination of these. Fracture and fragmentation are the focus of impact dynamics research. Impact causes the most serious damage in the form of holes and cracks, which can impair the material’s strength. Since fibre breaks in the impact contact zone, the residual tensile strength also drops significantly.

Delamination, a microscopic process that is difficult to identify by visual inspection but reduces residual compressive strength, is another outcome of impact damage [161]. It occurs exclusively at the intersections of layers with differing fibre orientations. Table 10 shows the damage caused by impact load in the previous study.

The shattered surface of kenaf/glass fibre-reinforced composites was studied by Malingam et al. Microcracks, fibre pull-out, fibre breaking, fibre bridging, debonding, and delamination at the interface were the most common damage types of materials in low-velocity impact tests [137]. Papa et al. alternatively stacked hybrid reinforced laminates with flax and basalt twill layers to investigate their dynamic behaviour. The findings of the experiments revealed that fibre hybridisation has a beneficial effect on damage [162]. Rajaei et al. evaluated the effects of heat-induced damage on the impact performance of epoxy laminates with glass and flax fibres. Heat convection (using a fan-assisted furnace) and impact properties partially damaged the composite laminates [176]. Ali et al. used the drop weight method to analyse the impact response of flexural behaviour of carbon/jute epoxy composites. The fracto-graphic analysis also showed failure modes. Increases in jute% resulted in higher damage area in drop weight impact testing [163]. Barouni and Dhakal characterised the damage investigation and assessment due to low-velocity impact on flax/glass hybrid composite plates. With the use of glass fibre as a hybrid reinforcement, the impact damage characteristics of flax natural fibre composites, such as impact load capability and absorbed energy, significantly improved [164]. The design and characterization of hybrid hemp/carbon laminates with improved impact resistance were evaluated by De Fazio et al. Non-destructive tests on the hybrid composite revealed a lesser extension of the damaged region (40% at 10 J and 10% at 20 J) confined at the interface between hemp and carbon fibres at the same absorbed energy level [165].

**Figure 4:**
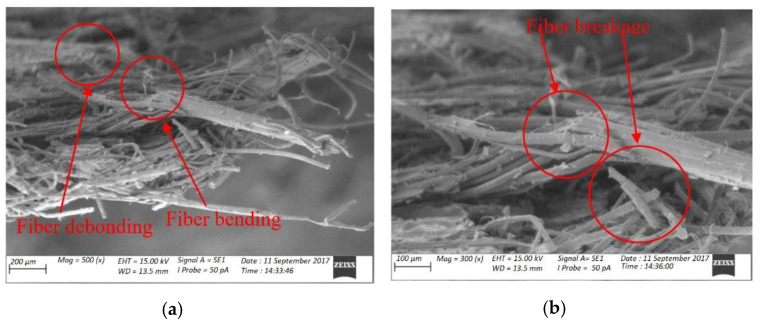
SEM images of fracture surface morphology of plain flax composites failed under impact loading at different magnifications (**a**) fibre debonding and bending; (**b**) fibre breakage reproduced from [167] MDPI, 2019.

**Figure 5:**
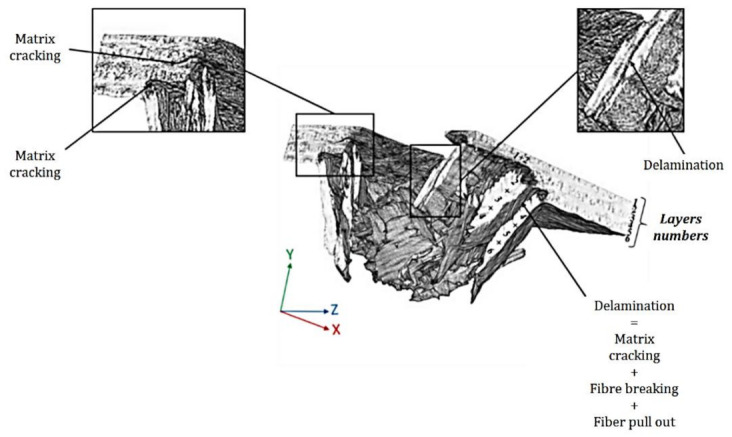
Micro CT scan image of a flax composite specimen (3/4 of the impact hole) after an impact test, with a 50 J impact energy, showing different failure modes in the specimen reproduced from [169] MDPI, 2020.

Sarasini et al. studied the hybridisation of basalt/flax fibres reinforced with polypropylene and epoxy. The ductile reaction to impact loads may be seen in the results. With the existence of matrix cracks and flax fibre failures on the impacted side, an extensive plastic deformation and a larger damaged area may be easily observed [166]. The effects of moisture exposure and elevated temperatures on the impact response of Pennisetum purpureum/glass-reinforced epoxy (PGRE) hybrid composites were evaluated by Ridzuan et al. They concluded that by increasing temperatures, the stiffness of the composites was shown to drop significantly, increasing the absorbed energy and peak deflection, causing serious damage to the specimens. Water immersion, on the other hand, did not result in a reduction in the impact load or energy absorption in a research on falling weight impact damage characterization of flax and flax basalt vinyl ester hybrid composites [168]. Chapman and Dhakal investigated the low-velocity impact and flexural capabilities, as well as damage characteristics, of flax-carbon/epoxy hybrid composites that might be employed in structural lightweight applications. The hybrid composite exhibited similar impact properties to plain carbon/epoxy composites in the damage analysis, greatly outperforming the performance of simple flax/epoxy composites alone. Figure 4 displays scanning electron microscopy (SEM) pictures of broken surfaces following the impact of plain flax/epoxy composites, which reveal significant fibre breakage and disorder, with one big group of fibres serving as an initial focal point. The following magnification scales (150 and 300) revealed matrix cracking and epoxy debonding from individual fibres, as well as fibre bending and debonding around a kink band in the flax fibre structure, with evident twisted and flattened fibres [167]. This was similar to the failure modes done by Dhakal et al., as in Figure 5.

In the study done by Hassan et al., the optimal banana/epoxy structure showed the most delamination and core breakage compared to the glass/epoxy system. Shear cracking predominated in the Kevlar, carbon, and glass composites under the CAI testing matrix [172]. Boria et al. performed analytical modelling and experimental validation of the low-velocity impact response of hemp and hemp/glass thermoset composites by varying fibre volume fractions. Six types of stacking sequences were used in the study of the hybrid effects of basalt and Kevlar fibres on the low-velocity impact behaviour of epoxy-based composites. According to the findings, the hybridisation of Kevlar fibres with basalt fibres increased the damaged area. The intercalated laminates had the greatest amount of damage and the least amount of penetration [170]. On the front and back surfaces of the fracture composite laminates, the damage behaviours of non-hybrid and hybrid PALF/Kevlar reinforced composites were studied by Feng et al. The back surface of the composite laminates showed more severe damage than the indented surface, showing that the composite laminates’ rear surface was more vulnerable to damage and distortion during the indentation process. The damage due to the indentation force can be due to the tension-shear and compression-shear. The damage began with a dent on the indented side, followed by crack start and propagation on the rear surface during the indentation test. The crack propagated continually as the indentation displacement increased until the composite laminates were completely fractured. The composite laminates showed fibre pullout, fibre matrix delamination, and fibre breaking [146].

The mechanical behaviour of an epoxy composite reinforced with Kevlar plain fabric and a glass/Kevlar hybrid fabric was investigated by Valenca et al. After Kevlar is replaced with glass fibres, the hybrid effect improves mechanical strength, as well as bending and impact energy [177]. A visual inspection done by Najeeb et al. revealed that the damaged area increased with an increase in the glass fabric in a drop weight test. According to the visual inspection acquired by the photographic image, the existence of woven fibre glass mat in the palf hybrid of glass fiber/palf/glass fibre (GPG) presented a different damage mechanism than palf. Furthermore, CT scan data revealed extensive interior damage in all damaged composites at the cross-section [174]. Hoekstra et al. examined the effects of machining processes on the damage response and surface quality of open-hole hybrid carbon/flax composites. Furthermore, delamination at the hole’s entry and departure, as well as secondary delamination, had a significant impact on the laminates’ damage progression [175].

## 8. Applications

In current and future innovation programmes, hybrid composite materials play a critical role. Hybrid composites are created by combining different fibres and matrix materials to achieve the desired qualities. Hybridization opens new possibilities for enhancing the toughness and impact resistance of composite materials, especially in advanced applications. In comparison to non-hybrid composites, hybrid composites offer more design freedom, resulting in a synergetic effect that no single material can achieve alone. The synergetic effect can be produced by a variety of factors, including fibre selection, fibre combination, and fibre interaction in the hybrid system. The features and properties of the materials must be understood by designers in order to create and handle them. The materials’ limitations must also be investigated, as hybridization may cause the composites’ performance to deteriorate [178].

### 8.1. Aerospace

Composite materials are now the material of choice for aerospace structural applications owing to their high specific strength and stiffness. Wings, control surfaces, and flow fan blades are all made from thin plate structures in aerospace applications [179]. Traditionally, the thermal, power, propulsion, and vehicle systems of aircraft have been built and optimized at the subsystem level, with little consideration given to the thermal management system. Because of the low thermal resistance of the airframe skin, the installation of ram inlet heat exchangers, and the comparatively modest amount of power required by the electrical loads, the design concept was sufficient. These considerations have resulted in the contemporary aircraft’s present thermal problems [180]. Ashikhmina et al. studied the optimal design methodology for suborbital tourist class reusable space vehicle (RSV) TC wing structures. The primary concerns in the RSV TC design, according to them, are weight efficiency, high service security, and the demand for economic feasibility of the RSV. This study implemented a multiscale modelling approach covering the design of hybrid composite material thermal properties (for example, at the mesoscale level), determination of thermal loads and thermal state analysis of a composite wing (i.e., macroscale and structural scale levels). Analysis of thermal fields demonstrated the necessity of thermal protection (TP) application for the wing structure. Consequently, optimal from the weight efficiency point of view, TP thicknesses were determined [181]. 

Safety and weight is the main focus for aircraft [163]. Light weighting design is a widely researched and applied idea in a variety of sectors, particularly in aircraft applications, and is linked to the green aviation co-opted concept. Reducing the mass of the aircraft, which needs less lift force and thrust during flight, is an efficient way to improve energy efficiency and minimize fuel consumption. In addition to lowering carbon emissions, lightweight design may improve flying performance by allowing for faster acceleration, increased structural strength and stiffness, and improved safety [182]. Chinvorarat studied the applicability of kenaf-based hybrid composite for aircraft radome. The radome of an aeroplane is a dome-shaped structure that protects radar antennas from dynamic stress, the environment, and impacts like bird attacks. They fabricated radome structure by using kenaf/glass reinforced epoxy through hand-layup techniques. They studied hybrid composite structures and discovered that kenaf/glass hybrid composites had a lot of promise to replace metal radomes [183]. In addition, the metallic impeller fan blades are instead fabricated with glass fibre reinforced plastics (GFRP). Characterization of GFRP blade material with partial jute reinforcements (glass-to-jute weight percentage of 95:5) was done, whereby properties like tensile strength, bending strength, shear strength, fracture toughness was investigated. By placing jute layer at various positions in GFRP blade material the mechanical strengths were estimated and compared with the conventional one [184]. Based on the findings, the optimal layup sequence for GFRP blades with partial woven jute reinforcements was selected, which has no significant impact on tensile parameters but improves biodegradability after service life. Metal alloys can be replaced with glass hybrid composites to lessen environmental impact and save money.

### 8.2. Automotive

In automotive, hybrid composite materials can be used to replace the majority of vehicle parts in order to improve their structure and performance. Effective weight reduction of existing metal/steel components in a car without sacrificing its strength would reduce fuel consumption, which will assist to minimize pollution. Many car manufacturers applied the hybrid composite in their car parts such as door panels, seat backs, windshield dashboard, boot-liner, instrument panel, gear and etc. [185]. In automotive applications, for example, hybrid composites are involved in the production of gear. Along with their low noise, low wear, self-lubrication, low weight, simple design, and manufacturing qualities, plastic gears are continuing to replace metal gears in vehicles, appliances, and machines. Because of several advantages, such as practically quiet movement, light weight, corrosion resistance, reduced friction coefficient, and the ability to run without external lubrication, the use of polymer gears is challenging. Temperature is another important factor that impacts the gear tooth [186]. Rana et al. designed and developed epoxy hybrid composite gears for low-powered applications. Here, hybrid composites are created by mixing different silicon oxide (SiO_2_) weight percentages with two layers of glass fibre simultaneously. The epoxy composites are selected for the development of spur gear pairs designed to have better mechanical strength and other valuable properties. The designed parameter stated was “Power: 2.5 HP Speed: 1000 RPM (Max)” [187].

Abdul Wahab et al. described the conceptual design process for a glass/renewable natural fibre reinforced polymer hybrid composite motorbike side cover. Motorcycle side covers are often constructed of plastic or steel, and they serve to protect motorcycle parts, components, and systems such as the frame, battery, electrical systems, and mechanical systems from damage. In the project, a glass/coir fibre-reinforced polypropylene hybrid composite material was chosen as an alternative to ABS for the motorbike side cover to improve environmental characteristics by utilizing agricultural waste. The usage of coir fibre makes the hybrid composite material partially biodegradable. Despite the fact that glass and coir fibres are denser than ABS, they can be compensated for by using polypropylene and reducing the amount of raw material used [188]. 

The mechanical characteristics of sisal/jute hybrid polymer composite qualities are evaluated and simulated in contrast to a model metal steel automotive chassis panel. This research compares the strength of sisal/jute fibre reinforced polyester-based hybrid composites to mild steel. The simulation findings for tensile and compressive strength were quite close to the experimental values. The mass density of the sisal jute composite was 1400 kg/m^3^, compared to 7858 kg/m^3^ for mild steel, which is a significant advantage in terms of weight, which aids their employment in vehicle bodies [189].

### 8.3. Defense

Military helmets and bulletproof jackets are manufactured as part of the defence development process, making the user safer [190]. Due to the expensive expense of aramide fibres and the need for an ecologically friendly replacement, a portion of the aramid was replaced with plain woven kenaf fibre, which came in a variety of thicknesses and configurations. The mechanical characteristics and ballistic performance were considerably impacted by the stacking sequence, thickness, and kenaf fibre content, according to the findings. By combining aramid and kenaf fibres, it was feasible to reduce the number of aramid fibres in a typical PASGT (Personal Armour System Ground Troops) shell by 12%, resulting in low-cost alternatives [191]. Azmi et al. looked explored the tensile, flexural, and high-velocity impact capabilities of a novel composite material that might be used to replace the present insert plate for bulletproof vests or body armour. A natural fibre (kenaf) and X-ray film were employed in this design. The materials were produced into several configurations with seven layers each using the typical hand lay-up process. The material was able to bounce back bullets launched at speeds of up to 105 m/s, indicating that the designs allowed it to absorb some impact energy. However, because ballistic testing utilise bullets that travel faster than high-velocity impact tests, the materials must still be improved before they can be used as a material for ballistic-resistant panels [192]. For similar purposes, Petre et al. studied novel polyurea-based composite materials (PUCs) and fibre-reinforced polymer composites for military applications, such as reducing blunt trauma for ballistic protection equipment in terms of thermal and mechanical properties and ballistic protection. The results indicated that PUCs can be successfully used for the fabrication of individual protection equipment [193].

## 9. Future Directions

In recent decades, researchers have been interested in hybrid composites from an environmental and economic standpoint. A proper selection of the constituents of the hybrid composite, as well as their characteristics, must be thoroughly studied to achieve better structural design with eco-friendly composites. The advantages of these hybrid composites, including reusability, environmental safety, and economic effectiveness, have made them a better substitute for typical synthetic materials, which may be used in a variety of advanced structural elements [194]. In wind turbines, tension-loading circumstances such as flexure and compression are important. Fatigue caused by blade flexure is the most common loading scenario. More extensive experimental and numerical studies of all of those features, but especially fatigue, will be required to advance the usage of fibre-hybrid composites in the wind energy industry [195]. 

Understanding the constituents’ significant material qualities, as well as the basic structures and manufacturing technologies, is required for the application of fibre-reinforced composites (FRPCs) in a range of sectors. For example, to make nanocomposites, one must first obtain nanotechnology, which includes all of the necessary tools and equipment. Furthermore, the manufacturing technique chosen has an impact on the final quality of the material. Understanding the constituents’ significant material qualities, as well as the basic structures and manufacturing technologies, is required for the application of fibre-reinforced composites (FRPCs) in a range of sectors. For example, to make nanocomposites, one must first obtain nanotechnology, which includes all of the necessary tools and equipment. Furthermore, the manufacturing technique chosen has an impact on the final quality of the material [196]. Hybrid composite materials, it should be noted, have revolutionized real-world commercial applications such as biomedical, aircraft, shipping, vehicles, biomedical, construction, drug delivery, wound dressing, and gas filtration. Changes in manufacturing techniques and the addition of nanofillers may lead to improvements in the future, as well as the ability to forecast results, attracting researchers to employ it in a variety of applications [197].

In order to maximize the benefits of hybrid strategies, which include both capsule- and fiber-based approaches, more research is needed. Self-healing composites made up of fast-healing capsules and small self-healing core-shell nanofibres, for example, can be utilized to repair damage in a wide spectrum of fissures, including ones as small as a few nanometers (owing to the nanofibres) [198].

## 10. Conclusions

Natural fibre reinforced polymer composites have advantages over synthetic composites in terms of low density and cost, making them excellent for commercial applications. Natural fibres have a positive influence on polymer mechanical performance when used as reinforcement in polymeric composites. This paper evaluates natural and synthetic fibers. The mechanical characteristics and low density of fibres are influenced by cellulose, hemicellulose, and lignin. Researchers have created novel hybrid reinforcements to overcome the limits of a single-fiber reinforced matrix due to the disadvantages of natural fibres. As a result, blending natural and synthetic fibres can improve the mechanical performance. Hybrid composites are studied for their physical, mechanical, thermal, and perforation resistance. Impact, flexural, and tensile strength were examined as essential factors that govern the mechanical behaviour of hybrid polymer composites. Such behaviour is influenced by the type, orientation, and arrangement of fibres in polymer composites. As can be observed, the mechanical characteristics of the hybrid fibres are equivalent to those of synthetic fibre composites. Natural-synthetic hybrid composites were also discussed in terms of their applications and future directions in many sectors.

## Figures and Tables

**Table 1:** Classifications of natural fibers. Adapted from [16] with permission from Elsevier, 2022.

**Natural Fibre**	Animal	Animal Hair	Wool, human hair, feather
		Silk	
	Mineral	Asbestos	Amosite, crocidolite, Tremolite, Actinolite, Anthophyllite
	Plant	Bast Fibre	Flax, Ramie, Hemp
		Leaf Fibre	Sisal, Pineapple
		Seed Fibre	Cotton
		Fruit Fibre	Coir
		Stalk Fibre	Rice

**Table 2:** The chemical composition of natural fibres. Data obtained from [25] Elsevier, 2021.

Natural Fibre	Cellulose	Hemicellulose	Lignin	Ash	MC	References
Banana	60–65	6–8	5–10	2.7–10.2	10–15	[26]
Cotton	89–96	2.3	0.2–0.5	0.6–1.5	0.5–0.8	[27]
Bamboo	73.83	12.49	10.15	9.6	3.16	[28]
Bagasse	55.2	16.8	25.3	1.5–5	8.8	[29]
Hemp	68	15	10	0.8	6.2–12	[29]
Kenaf	45–57	21.5	8–13	-	-	[30]
Pineapple	70–80	18.8	12.7	0.9–1.2	11.8	[30]
Flax	71	18.6–20.6	2.2	-	8–12	[29]

**Table 3:** Summary of natural fibre properties from researchers.

Natural Fibre	Density (g/cm^3^)	Tensile Strength (MPa)	Elongation (%)	Elastic Modulus (GPa)	References
Banana	1.35	529–914	2.6–5.9	27–32	[35]
Cotton	1.51	400	3–10	12	[36]
Bamboo	1.5	575	3	27	[37]
Bagasse	1.25	290	2.11	11	[38]
Hemp	1.47	690	2.38	70	[39]
Kenaf	1.45	930	1.6	53	[40]
Pineapple	1.5	900–1600	3.0	70–82	[41]
Flax	1.4	1400	1.6	70	[42]
Jue	1.5	393–1000	2.5	13–54	[43]
Sisal	1.33–1.5	80–855	2.14	9–22	[44]

**Table 4:** The advantages and the disadvantages of fully green composites over conventional petrochemical-based composites [46] with permission from Faculty of Design and Technology of furniture and interior, 2017.

Advantages	Disadvantages
Renewable resources	Inhomogeneous structure of fibres
Lower production costs	Dimensional instability as a negative consequence of water absorption
Good specific mechanical properties	Lower water and thermal resistance
Lower density of composites	Susceptibility to microbial attacks and rotting
Reduced energy consumption during manufacturing	Insufficient adhesion and incompatibility with the polymer matrix
Biodegradability and eco-friendly materials	Degradation and aging
Lower risk to human health	Restricted processing temperature (to avoid thermal degradation)

**Table 5:** Properties of glass fiber, carbon fiber, and Kevlar. Adapted from [49] with permission from Chaoyang University of Technology, 2017.

Synthetic Fibers	Density (g/cm^3^)	Tensile Strength (MPa)	Tensile Modulus (GPa)	Elongation (%)
Aramid	1.4	3000–3150	63–67	3.3–3.7
Glass fiber				
E-glass	2.5	2000–3500	70	2.5
S-glass		4570	86	2.8
Carbon fiber	1.4	4000	230–240	1.4–1.8

**Table 6:** Thermal studies from several studies.

Hybrid Fibre	Thermogravimetric Analysis (TGA)			Differential Scanning Calorimetric (DSC)		References
	Initial Degradation Temperature IDT (°C)	Final Degradation Temperature FDT (°C)	Final Residue (%)	T_g_	T_d_	
Pennisetum purpureum/glass	76.30–121.10	440–534	1.6–22.9	64		[71]
Cocos nucifera/glass	100–150	500	11–24	70–80	350–400	[72]
Sugar palm/glass	299–340	360–400	7–16	82.50	80–130	[73]
Sugar palm/glass	138–156	440–534	5			[74]
Jute/glass	270–300	300–336		100		[75]
Kenaf/Carbon	341334	315–390	13–54	100		[76]
Kevlar/jute/flax/hemp/sisal	210	340	30–51	90–105	400	[77]
Jute/Glass	318–390	437–439	6–46			[78]
Flax/Carbon		600	41	80	240	[79]

**Table 7:** Research works reported on mechanical properties of natural-synthetic hybrid composites.

Matrix	Fiber	Parametric	Tensile Strength (MPa)	Tensile Modulus (GPa)	Flexural Strength (MPa)	Impact Strength (k/Jm^2^)	References
Epoxy	Glass-Basalt/Flax/Jute	Fiber loading	450	9.20	410	-	[91]
Epoxy	Banana/carbon	Hybridization Water absorption	277	-	307	-	[84]
Polyester	Kenaf/rGlass	Hybridization	-	-	181.98	-	[92]
Epoxy	Kenaf/Aramide	Hybridization Fiber loading	114.49	1.75	-	-	[93]
Polyester	Kenaf/Glass	Hybridization Weathering effect	70	3.0	120	-	[94]
Epoxy	Palf/glass Coir/glass	Fiber loading Water absorption	52	2.10	120	-	[95]
Epoxy	Banana/Palf/Glass	Fiber loading Thermal	132.29	11.52	-	-	[73]
Polyester	Glass/Jute	Fiber loading	78.61	4.26	146.30	45	[96]
Epoxy	Aramide/Coir	Fiber loading	-	-	-	149	[97]
Epoxy	Glass/Kenaf	Fiber loading	175.68	1.72	-	-	[98]
Epoxy	PALF/Carbon	Fiber loading Water absorption	43.13	1.86	-	-	[85]
Epoxy	PALF/Glass	Fiber loading Water absorption	40.43	2.40	171	-	[99]
Epoxy	Jute/Carbon	Fiber loading, Thermal, Water absorption	301	-	-	127	[100]
Epoxy	Jute/Carbon	Fiber loading	257.60	9.80	-	-	[101]
Epoxy	Carbon/Jute/Banana	Fiber loading	160	380	-	-	[102]
Epoxy	Aloevera/ Bamboo/Palm/Kevlar	Hybridization	123	210	-	-	[103]
Epoxy	PALF/Carbon	Fiber loading	187.67	7.87	247.61	-	[104]
Epoxy	Kevlar/Aloe Vera/Bamboo	Fiber loading	127	298.38	223.48	-	[105]
Epoxy	Date Palm /Kevlar	Fiber loading	237	3.60	-	-	[106]

**Table 8:** Difference of Charpy and Izod Test adapted from [114] with permission from Akademia Baru, 2017.

Type of Test	Izod	Charpy
Specimen Position	Vertical	Horizontal
Point of Strike	Upper tip of specimen	Point of notch but in opposite direction
Direction of Notch	Facing the striker that is fastened to the pendulum	Away from striker
Type of Notch	V-notch	V-notch and U-notch
Type of Hammer	Farming hammer	Ball in hammer

**Table 9:** Reported research on perforation resistance of hybrid composite laminates.

References	Hybrid Composite	Parameters	Low-Velocity Impact Performance		Remarks
			Peak Force (kN)	Energy Absorbed (J)	
[63]	Kevlar Basalt	Stacking sequence	5.04	70.60	Alternative stacking of basalt and Kevlar fabrics enhanced 15.58–20.79% and 13.47–20.47% improvement in the peak force and energy absorption.
[132]	Kenaf glass	Clamping conditions			Natural frequency decreases with increasing impact level.
[133]	Jute Basalt	Environmental conditions	4.00	7.71	Result exhibited a higher degree of strength retention after environmental aging, thus confirming the positive role played by basalt fibres in enhancing the durability of natural fibre composites
[91]	E-glass Jute Basalt Flax	Fibre loading	3.00	30.00	Impact test showed a higher peak force while maximum deflection was governed by indentation test for hybrid and homogeneous composites
[134]	Flax Basalt	Environment conditions	-	-	Did not exhibit such a large difference in impact behaviour between dry and conditioned
[135]	Flax Basalt	Stacking sequence	-	-	The more complex structure presented by the hybrid, including two materials with different strength, is likely to reduce the extent of the striker rebound
[136]	Carbon Glass	Stacking sequence	3.94	19.24	Pipes with CGG stacking represents higher impact resistance while the GCG stacking has a better response of damage formation since this stacking does not show leakage damage.
[137]	Kenaf Kevlar	Fiber configurations	4.00	30.00	The bending stiffness of each fibre ply determines the penetration resistance of composite laminates. As a result, the inclusion of Kevlar fibre in the surface layers increased the laminates’ penetrating resistance.
[138]	Aramid Kenaf	Stacking sequence	44.63	409.70	The composites exhibited a larger effective displacement for complete penetration because of the visco-elastic-plastic behaviour of the polypropylene system
[139]	Kenaf Jute glass	Stacking sequence	6.20	30.00	Alternate sequence of hybrid composite exhibits more impact resistance
[140]	Carbon Flax	Stacking sequence	-	-	Composite with alternate sequence exhibit lower absorbed energy, higher penetration energy, smaller crack lengths, smaller indentation depths, smaller damage areas, lower temperature rise, and higher impact strength.
[5]	Glass Kenaf	hybridization	2.60	-	Impact peak force and displacement increase with energy level
[141]	Carbon Glass Basalt	Fiber configuration	9.17	59.43	Carbon fibre as the core exhibited superior impact resistance and weave fabric composite of basalt fibre laminates exhibited better energy absorption capability and deformation resistance
[142]	Flax Carbon Glass MWCNT	Nanofiller enhancement	2.00	13.18	The value of absorbed energy for carbon/flac was higher compared to that of glass/flax due to severe damage occurred on carbon/flax surface compared to that of glass/flax.
[143]	Aluminium Glass Carbon	Stacking sequence	6.70	87.61	Hybrid laminates shows 15% improvement of energy absorption and glass plies in H1 hybrid FMLs were able to distribute the contact stress, while the middle layer acted as a barrier in resisting crack propagation.
[144]	Carbon Flax	Stacking sequence	5.80	-	Compared to a non-hybrid flax composite of same thickness, flax plies on the affected side result in a considerable improvement in impact resistance
[145]	Flax Basalt	Stacking sequence	3.10	-	Due to the interlaminar strength of the fibres, alternate layers of basalt and flax fibres suffered less damage.
[146]	PALF Glass	Stacking sequence/hybridization	1.47	15.10	Glass fibre was partially included into the composite laminates, which increased indentation resistance and energy absorption.
[147]	Banana Glass	Stacking sequence	4.69	27.12	The addition of glass fibre to a banana fibre reinforced composite improves energy absorption and overall impact performance.
[148]	Kenaf Glass	Fibre loading	9.31	23.23	The hybrid composites can endure up to 40 J of impact energy, with the peak impact load and absorbed energy increasing as the incident impact energy increased.
[149]	Bamboo glass	Nanofiller enhancement	-	-	CNTs absorbed less energy than bamboo/glass hybrids without them, resulting in less physical damage.
[109]	Flax Carbon	Fibre configuration	2.41	19.94	When compared to 5 carbon layers, the energy absorption of the hybrid composites rises by 13.25 percent for the FCFCF sample and 28.89 percent for the CFFFC sample.
[150]	Jute Glass	Stacking sequence	5.60	46.89	In comparison to composites with glass fabric layers in the inside and flax or jute textiles, hybrid composites with glass fabric layers on the exterior had greater impact resistance.
[151]	Flax Glass	Fibre loading	-	-	Hybridization of glass fibre onto flax fibre composite improves impact damage characteristics by generating a balanced effect.
[152]	Jute Kevlar	Impactor height	0.22	4.20	The dynamic reaction of these frameworks relies upon the flexible properties of the fibre material
[153]	Bamboo Glass	Hybridization	6.10	27.92	Increased filler loading reduced the severity of damage in non-hybrid composites, while the addition of woven glass fibre slowed the impactor’s penetration, lessening the risk of total failure.
[154]	Oil palm EFB Kevlar	Stacking sequence	5.00	30.00	The layering sequence K/OP/K in Kevlar/OPEFB hybrid composites can resist up to 35 J of impact energy, with the optimal gamma radiation dosage at 50 kGy
[155]	Flax Basalt	Energy level, temperature, and number of impacts	5.81	-	Decreasing temperatures caused an embrittlement effect on neat PP composites with an increase maximum force and a decrease of maximum displacement, whereas increasing temperatures led to a softening of compatibilized composites with a decrease of maximum force and an increase maximum displacement
[156]	CFRP Wood	Fibre loading	3.50	-	Wood cells deform during impact and hence dissipate more energy

**Table 10:** Damage caused by impact load of hybrid composites.

References	Hybrid Composite	Parameters	Failure Modes
[137]	Kenaf Glass	Fiber configuration	Microcracks, fiber pull out, fiber breakage, fiber bridging, debonding, and delamination at the interface.
[162]	Flax Basalt epoxy	Hybridization	Matrix cracking, delamination, propagation and fibre failure
[162]	Glass Flax epoxy	Temperature exposure	The heat exposed specimen indicates the delaminated regions and/or fibre-matrix separation.
[163]	Carbon Jute epoxy	Fiber configuration	Matrix and fiber failure, ply failure, fiber pullout, delamination.
[164]	Flax Glass VE	Hybridization	Matrix cracking, delamination, fibre breakage and, finally, penetration
[165]	Hemp Carbon epoxy	Fiber configuration	Localized buckling, fiber breakage, matrix cracking
[166]	Flax Basalt PP/Epoxy	Matrix hybridization	Shows ductile response to impact loading. An extensive plastic deformation and a wider damaged area can be easily observed with the presence of matrix cracks and flax fiber failures on the impacted side.
[167]	Carbon Flax epoxy	Fiber configuration	Fiber debonding, bending, fiber breakage
[168]	Pennisetum purpureum Glass epoxy	Environment ondition	Matric cracking, delamination and fiber breakage. The damage pattern was more visible and extended at higher temperatures
[169]	Flax Basalt VE	Environment conditions	Matrix cracking, delamination, fibre breakage, and fibre pull out.
[170]	Hemp Glass epoxy	Fiber configuration	Delamination, fiber fracture, matrix cracking
[146]	PALF Kevlar PP	Fiber configuration	Crack initiation and propagation, fiber pull-out, fiber- matrix delamination and fiber breakage were
[171]	Basalt Kevlar Epoxy	Fiber configuration	Matrix cracking, delamination.
[172]	Banana Carbon Kevlar epoxy	Hybridization	Matric cracking, delamination and fiber breakage corresponds to delamination of the skin and brittle fracture of the core
[173]	Kevlar Glass epoxy	Fiber configuration	Delamination. The damaged area is increased with an increase in the glass fabric percentage.
[174]	PALF Glass epoxy	Fiber configuration	Matrix crack, fiber breakage. Presence of glass fiber increase the elasticity of composite and the damage clearly visible.
[175]	Carbon Flax epoxy	Fiber configuration	Fiber matrix debonding and fiber breakage

## Data Availability

Not applicable.
